# Prediction of disease severity using serum biomarkers in patients with mild-moderate Atopic Dermatitis: A pilot study

**DOI:** 10.1371/journal.pone.0293332

**Published:** 2023-11-02

**Authors:** In-Seon Lee, Mijung Yeom, Kyuseok Kim, Dae-Hyun Hahm, SeHyun Kang, Hi-Joon Park

**Affiliations:** 1 College of Korean Medicine, Kyung Hee University, Seoul, Republic of Korea; 2 Acupuncture & Meridian Science Research Center, Kyung Hee University, Seoul, Republic of Korea; 3 Department of Ophthalmology, Otorhinolaryngology and Dermatology of Korean Medicine, College of Korean Medicine, Kyung Hee University, Seoul, Republic of Korea; 4 College of Medicine, Kyung Hee University, Seoul, Republic of Korea; University of Missouri, UNITED STATES

## Abstract

Atopic dermatitis (AD) is an inflammatory skin condition that relies largely on subjective evaluation of clinical signs and symptoms for diagnosis and severity assessment. Using multivariate data, we attempted to construct prediction models that can diagnose the disease and assess its severity. We combined data from 28 mild-moderate AD patients and 20 healthy controls (HC) to create random forest models for classification (AD vs. HC) and regression analysis to predict symptom severities. The classification model outperformed the random permutation model significantly (area under the curve: 0.85 ± 0.10 vs. 0.50 ± 0.15; balanced accuracy: 0.81 ± 0.15 vs. 0.50 ± 0.15). Correlation analysis revealed a significant positive correlation between measured and predicted total SCORing Atopic Dermatitis score (SCORAD; r = 0.43), objective SCORAD (r = 0.53), eczema area and severity index scores (r = 0.58, each *p* < 0.001), but not between measured and predicted itch ratings (r = 0.21, *p* = 0.18). We developed and tested multivariate prediction models and identified important features using a variety of serum biomarkers, implying that discovering the deep-branching relationships between clinical measurements and serum measurements in mild-moderate AD patients may be possible using a multivariate machine learning method. We also suggest future methods for utilizing machine learning algorithms to enhance drug target selection, diagnosis, prognosis, and customized treatment in AD.

## 1. Introduction

Atopic dermatitis (AD) is a common yet complex inflammatory skin disease, characterized by intense itching and recurrent eczematous lesions [1]. To-date, successful control of AD symptoms has been challenging owing to its highly heterogeneous nature [2]. Additionally, the diagnosis and severity assessment of AD currently relies solely on subjective tools (e.g., patient self-reports, visual inspection by clinicians) with large inter- and intra-observer variability [3]. Therefore, attempts have been made to identify more reliable and objective indicators, known as biomarkers, that may reduce observatory differences, help with the diagnosis and evaluation of AD and its severity, and even potentially predict AD onset or treatment response [4].

Over recent decades, a number of biomarkers for AD, such as immunoglobulin E (IgE; [5,6]), and thymus and activation-regulated chemokine (TARC/CCL17; [7,8]), have been found using conventional approaches. These approaches include univariate analyses, comparing properties of AD patients with those of healthy controls (HC), and correlation or regression analyses between potential biomarkers and symptom severity of AD. However, it is highly unlikely that one biomarker will be able to diagnose and assess the severity of AD in its entirety [9].

Recently, multivariate machine learning analysis has been used in many studies (e.g., for the study of pain [10,11]) to reveal hidden patterns between variables and to develop more powerful predictive models. Thijs et al. (2017) found that combining various serum biomarkers, such as TARC, IL-22, and sIL-2R, improved their model’s performance in predicting eczema area and severity index (EASI) scores compared to using a single biomarker. Additionally, combined biomarkers showed a better correlation coefficient with disease severity than did single biomarkers [9]. Ungar et al. (2017) tested various lesional and non-lesional biomarkers before and after cyclosporine A treatment in AD patients and found that the model that integrated non-lesional biomarkers showed better correlations with an improvement of SCORing Atopic Dermatitis score (SCORAD) [12]. More recent studies by Bakker et al. used serum biomarkers to predict EASI scores treated with duplimab [13] and to confirm their pre-defined biomarkers which can identify AD endotypes in a new patients with AD [14]. According to Hurault et al., adding biomarkers to the Bayesian state-space model for predicting AD severity scores did not improve model performance [15], indicating that additional studies are required to support the use of serum cytokines/chemokines as biomarkers for AD. Given that patients with mild AD account for as many as 27–67% of all patients [16], we also need a model for diagnosis and prediction of symptom severities in those patients, additional to the patients with severe symptoms.

In this study, we aimed to test a feasibility of developing a diagnostic tool and severity prediction model using multivariate machine learning technique for AD patients. We conducted phase I machine learning analysis, in which collection of multivariate data, splitting data into training and test data, model training, estimation of the prediction performance of the model, feature estimation, and feature selection were performed, combining clinical and serological markers from a previous clinical study [17,18].

## 2. Materials and methods

### 2.1. Participants

We used data from our previous study [17], in which the effect of acupuncture treatment was reported. This study was approved by the Kyung Hee University Korean Medicine Hospital Institutional Review Board (IRB No. KOMCIRB-160212-HRBR-004), and informed consent was obtained from all participants before the experiment. In brief, 30 mild-moderate AD patients and 20 HC participated. The diagnosis and severity rating of atopic dermatitis were made based on the criteria of Hanifin and Rajka and the objective SCORing Atopic Dermatitis score (SCORAD) index, respectively. Eligible patients were 19 years of age or older, with mild to moderate atopic dermatitis, as defined by objective SCORAD scores of 10–40 (for the detail selection criteria, see our previous report [17]). Healthy, age- and gender-matched HC were included for comparison. We measured demographics and clinical characteristics (e.g., AD severity score), cytokine/chemokines, cortisol, and total IgE. AD patients were randomly assigned to low-dose acupuncture, high-dose acupuncture, and sham acupuncture group, and 2 patients did not complete the study. Pre- and post-treatment data from 66 visits with 28 AD patients and 20 data points from 20 HC patients (all pre-treatment visits) were included.

### 2.2. Assessment of AD severity

AD severity was assessed using SCORAD index and EASI. The objective SCORAD score is based on the extent of disease and intensity of signs (investigator-assessed), and the total SCORAD score is calculated using the objective SCORAD score and two subjective symptom scores (pruritus and insomnia) [19]. Pruritus and insomnia were each rated by a 100-mm visual analogue scale (VAS). EASI is another effective tool for objectively assessing severity and extent of AD in four different body regions [20].

### 2.3. Serum sample collection and cytokine analyses

Peripheral blood samples from AD patients were obtained at baseline (week 0), on completion of the 4-week acupuncture treatment (week 4) and at the 4-week follow-up visit (week 8), and those from HC was obtained at baseline (week 0). Serum was separated by centrifugation (at 3,000 rpm for 10 min at room temperature) after clotting and stored at −80°C until use.

Serum cytokine and chemokine levels were measured using Bio-Plex Pro Human Chemokine 40-plex Panel (Bio-Rad Laboratories, Hercules, California) and Luminex 200 System (Luminex Corp., Austin, Texas) according to the manufacturer’s instructions. Measurements were performed in diluted samples (1:4). Concentrations of cytokines and chemokines were determined on the basis of the fit of a standard curve with four-parameter logistic regression for mean fluorescence intensity versus pg/mL using Bio-Plex Manager™ 6.1 software (Bio-Rad).

### 2.4. Measurements of total IgE and cortisol levels

Serum levels of total IgE and cortisol were measured with commercially available Human IgE ELISA Kit (Koma Biotech, Korea) and Corticosterone ELISA kit (Abcam, Cambridge, MA), respectively, according to manufacturer’s instructions.

### 2.5. Statistical analysis

#### 2.5.1. Univariate analysis

Continuous variables are presented as mean ± standard deviation (SD) for normally distributed data and median and interquartile range (IQR) for non-normally distributed data, and categorical variables as counts and percentages. Unpaired t-test was used when continuous variables were normally distributed. If data were not normally distributed, the Mann-Whitney U test was used. Chi-square test was used to compare categorical variables. Statistical analyses were performed by using GraphPad Prism 9.02 (GraphPad Software, San Diego, CA, USA). The statistical significance was set at *p* < 0.05 (false discovery rate corrected). 

#### 2.5.2. Multivariate machine learning analysis

Among various machine learning algorithms, we applied Random forest (RF), an ensemble learning algorithm based on bootstrap aggregation of multiple trees constituting the forest. Using randomForest package in R (version 4.0.0, http://www.r-project.org), we developed two RF models, one for classification and another for regression analysis. Missing cases were excluded for all analyses. For the classification model, we trained RF classifier on combinations of demographic variables (age, sex, body mass index [BMI], and smoking status), cytokine measurements, cortisol, and total IgE level to discriminate AD patients versus HC. For the regression model, RF classifier was trained on the same data to predict clinical outcomes such as total SCORAD score, objective SCORAD score, EASI, and itch ratings measured by visual analogue scale (VAS).

The performance of the classification model was tested using stratified 4-fold cross validation, and the whole cross validation procedure was repeated 1000 runs on 1000 different learning and test sets. The area under the curve (AUC) and balanced accuracy were estimated for each run to evaluate the classification performance. To determine whether the classification did not merely occur by chance, we also conducted a random permutation test for the classification model. We randomly permuted the labels of the Group (AD and HC) and run the same RF algorithm on the data with these random labels (*n* = 1000). AUC and balanced accuracy were assessed for each run, and we compared the AUC and balanced accuracy values of random permutation test to those of our models using two sample z-test. For the regression models, the performance of the regression model was assessed using mean squared error (MSE) and correlation analysis between predicted and measured scores. To find the most contributing factors among independent variables for both models, importance of each feature was evaluated using importance function in R (mean decrease in accuracy for the classification model and percent increased MSE for the regression model). The statistical significance was set at *p* < 0.05. 

## 3. Results

### 3.1. Participants characteristics

The sixty-six data of patients with AD and 20 of HCs were included and their baseline characteristics are shown in Table 1. There were no significant differences in demographic characteristics between AD and HC groups. Patients were diagnosed with AD according to the criteria of Hanifin and Rajka, and all had mild to moderate AD (objective SCORAD = 26.4 ± 6.3). In AD patients, the median (IQR) total SCORAD was 38.4 (33.0–41.3) and EASI was 4.2 (2.1–5.6), and VAS pruritus and VAS sleep loss were 6.0 ± 1.1 pg/ml and 3.9 ± 2.5 pg/ml, respectively. The AD patients showed significantly elevated total IgE level (median = 1199.4 IU/ml, IQR = 218.1–2569.2 IU/ml) compared to HC (122.1 [52.8–169.1] IU/ml; *p* < 0.001). Serum cortisol levels showed no significant difference between groups (*p* = 0.347).

**Table 1:** Baseline characteristics of participants.

	AD patients (n = 28)		Healthy controls (n = 20)		*p* value
	mean (SD)	95% CI	mean (SD)	95% CI	
**Age (years)**	22.89 (5.06)	20.93–24.85	23.25 (3.27)	21.72–24.78	0.369
**Gender (male/female, *n* (%))**	10 (35.7) /18 (64.3)		8 (40.0)/12 (60.0)		0.762
**BMI (kg/m2)**	22.44 (3.05)	21.25–23.62	21.55 (1.75)	20.73–22.37	0.21
**Total IgE (IU/ml)**	1409.2 (1264.44)	918.91–1899.5	123.63 (86.47)	83.16–164.1	<0.001***
**Cortisol (ng/ml)**	108.6 (49.99)	89.21–127.98	122.98 (56.14)	96.71–149.25	0.347
**Objective SCORAD**	26.38 (6.25)	28.8–23.95	-	-	-
**Total SCORAD**	36.14 (7.41)	39.02–33.27	-	-	-
**EASI**	4.34 (2.76)	5.41–3.27	-	-	-
**VAS itch**	6.03 (1.06)	6.44–5.62	-	-	-
**VAS sleep loss**	3.9 (2.46)	4.85–2.94	-	-	-

Results are presented as Mean, standard deviation (SD) and 95% confidence intervals (CI). Corresponding *p* values were obtained from the unpaired t test, Mann-Whitney U test and chi-square test, as appropriate. *** significantly different at *p* ≤ 0.001. AD, atopic dermatitis; HC, healthy controls; BMI, body mass index; SCORAD, scoring atopic dermatitis; EASI, eczema area and severity index; VAS, visual analogue scale; NA, not available.

### 3.2. Serum cytokine/chemokine levels

Total IgE levels and 27 variables among the 40 cytokines/chemokines assessed (BCA-1/CXCL13, CTACK/CCL27, ENA-78/CXCL5, Eotaxin/CCL11, Eotaxin-2/CCL24, Eotaxin-3/CCL26, Fractalkine/CX3CL1, GCP-2/CXCL6, GM-CSF, Gro-α/CXCL1, I-309/CCL1, IFN-ϒ, IL-1β, IL-2, IL-4, IL-6, IL-8/CXCL8, IL-10, MCP-3/CCL7, MCP-4/CCL13, MDC/CCL22, MIG/CXCL9, MIP-1α/CCL3, MIP-3β/CCL19, TARC/CCL17, TECK/CCL25, and TNF-α) showed a significant difference between AD and HC groups (S1 Table).

### 3.3. Classification of patients with AD from HC

**Fig 1:**
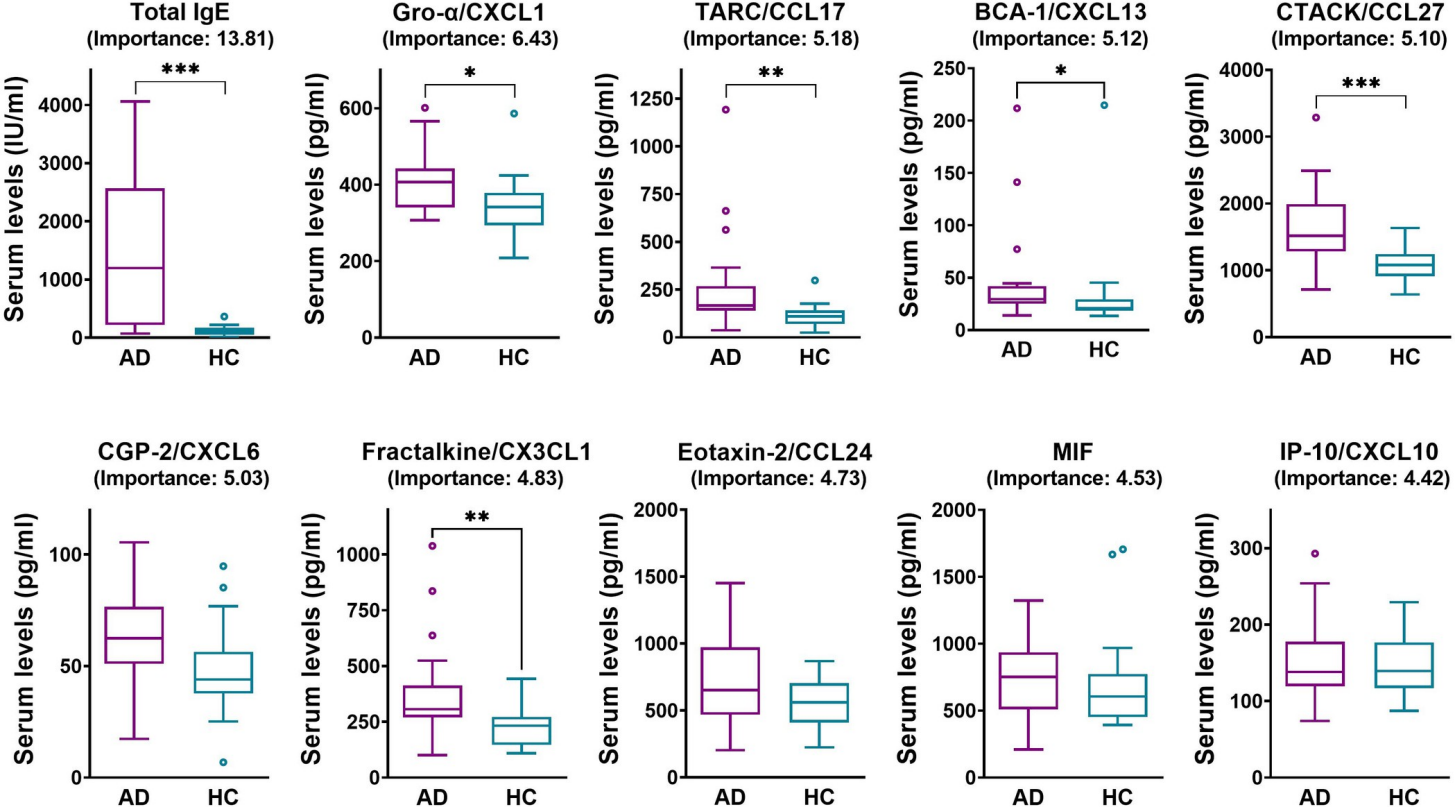
Serum levels of the top 10 important features selected by the random forest classification model for diagnosis in atopic dermatitis (AD) patients and healthy controls. Data are presented as box plots with Tukey whiskers. ns, *p* > 0.05; *p ≤ 0.05; **p ≤ 0.01; ***p ≤ 0.001 by unpaired t test and Mann-Whitney test as appropriate. AD, atopic dermatitis; HC, healthy controls.

Excluding missing data resulted in a final sample of 20 HC and 66 AD patients’ data (measured from 28 AD patients). Using all available predictors in a 4-fold cross validation model showed an AUC of 0.85 ± 0.10 and a balanced accuracy of 0.81 ± 0.15 (accuracy: 0.83 ± 0.08; sensitivity: 0.84 ± 0.05; specificity: 0.78 ± 0.28; F1: 0.89 ± 0.05; precision: 0.95 ± 0.07; recall: 0.84 ± 0.05). IgE (importance: 13.81), Gro-α/CXCL1 (6.43), TARC/CCL17 (5.18), BCA-1/CXCL13 (5.12), CTACK/CCL27 (5.10), GCP-2/CXCL6 (5.03), Fractalkine/CX3CL1 (4.83), Eotaxin-2/CCL24 (4.73), MIF (4.53), and IP-10/CXCL10 (4.42) were ranked as the most important features, as measured by mean decrease in accuracy for the classification, and their univariate analysis results are shown in Fig 1. Two-sample z-test revealed that both AUC and balanced accuracy of the model with correct label were significantly greater than those of random permutation model (permutation *n* = 1000; AUC: 0.85 ± 0.10 vs. 0.50 ± 0.15, z = 61.705, *p* < 0.001, 95% CI 0.34 ‐ 0.36; balanced accuracy: 0.81 ± 0.15 vs. 0.50 ± 0.19, z = 31.11, *p* < 0.001, 95% CI 0.29 ‐ 0.33).

### 3.4. Prediction of disease severity in AD patients

**Fig 2:**
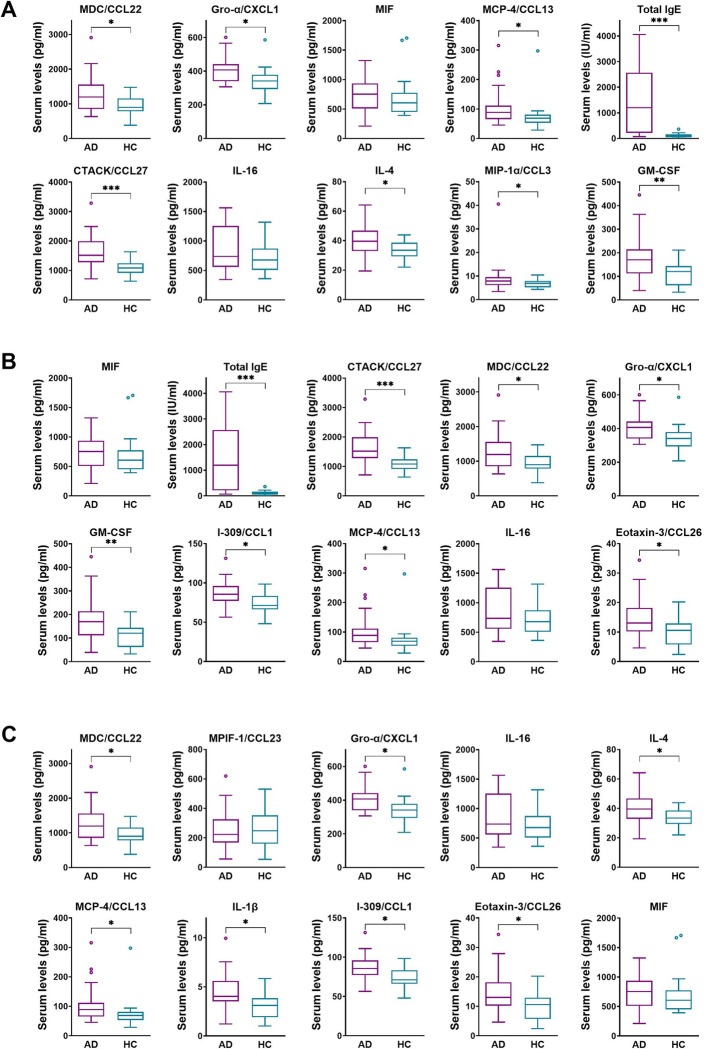
Serum levels of the top 10 important features contributing to each predictive random forest classification model for disease severity in atopic dermatitis patients and healthy controls. Random forest machine learning algorithms using serum cytokine/chemokine measures were used to predict the clinical outcomes, objective SCORAD (A), total SCORAD (B), and EASI scores (C). Data are presented as box plots with Tukey whiskers. ns, *p* > 0.05; *p ≤ 0.05; **p ≤ 0.01; ***p ≤ 0.001 by unpaired t test and Mann-Whitney test as appropriate. AD, atopic dermatitis; HC, healthy controls.

Excluding missing data resulted in a final sample of 66 AD data (measured from 28 AD patients). Using all available predictors, RF regression algorithm was used to predict total SCORAD, objective SCORAD, EASI, and VAS itch ratings. Test root MSE for objective SCORAD was 6.43 ± 0.90, for total SCORAD was 9.19 ± 1.24, for EASI was 1.83 ± 0.33, for VAS itch ratings was 2.03 ± 0.29. MAE for objective SCORAD was 6.43 ± 4.36, for total SCORAD was 9.20 ± 6.16, for EASI was 1.83 ± 1.47, for VAS itch rating was 2.02 ± 1.40. Correlation analysis showed significantly positive correlation between measured d objective SCORAD and predicted objective SCORAD scores (r = 0.53, t = 4.74, df = 64, p < 0.001, 95% confident interval [CI] 0.32 ‐ 0.69), between total SCORAD and predicted total SCORAD scores (r = 0.43, t = 3.73, df = 64, p < 0.001, 95% CI 0.20 ‐ 0.61), between EASI and predicted EASI scores (r = 0.58, t = 4.40, df = 64, p < 0.001, 95% CI 0.33 ‐ 0.76), and not significant correlation between VAS itch ratings and predicted VAS itch ratings (r = 0.21, t = 1.38, df = 66, p = 0.18, 95% CI -0.10 ‐ 0.49). Based on the percentage increase in MSE score, importance of each feature was determined (Table 2). We found that Gro-α/CXCL1, MCP-4/CCL13, MDC/CCL22, MIF, IL-16 were ranked all in top 10 in the prediction models for total SCORAD, objective SCORAD, and EASI scores. Total IgE level failed to rank in top 10 only in the prediction model for EASI score (univariate analysis results are shown in Fig 2).

**Table 2:** Top 10 important features for Random Forest prediction model.

Objective SCORAD	Total SCORAD	EASI	Itch (VAS)
**Feature (MSE)**	**Feature (MSE)**	**Feature (MSE)**	**Feature (MSE)**
MDC/CCL22 (6.07 ± 2.24)	MIF (7.05 ± 2.63)	MDC/CCL22 (7.41 ± 2.65)	MIF (9.08 ± 3.35)
Gro-α/CXCL1 (5.76 ± 2.22)	IgE (4.85 ± 1.86)	MPIF-1/CCL23 (6.10 ± 2.27)	CTACK/CCL27 (3.83 ± 1.67)
MIF (5.76 ± 2.03)	CTACK/CCL27 (4.74 ± 1.76)	Gro-α/CXCL1 (4.49 ± 1.49)	IL-1 (3.44 ± 1.53)
MCP-4/CCL13 (5.70 ± 1.99)	MDC/CCL22 (3.99 ± 1.39)	IL-16 (4.26 ± 1.34)	BCA-1/CXCL13 (3.15 ± 1.32)
IgE (5.50 ± 1.91)	Gro-α/CXCL1 (3.84 ± 1.38)	IL-4 (4.15 ± 1.21)	IgE (3.10 ± 1.30)
CTACK/CCL27 (3.82 ± 1.35)	GM-CSF (3.77 ± 1.27)	MCP-4/CCL13 (4.13 ± 1.14)	MIP-3Α/CCL20 (2.99 ± 1.27)
IL-16 (3.74 ± 1.22)	I-309/CCL1 (3.64 ± 1.24)	IL-1 (3.95 ± 1.12)	Eotaxin-2/CCL24 (2.89 ± 1.21)
IL-4 (3.61 ± 1.21)	MCP-4/CCL13 (3.53 ± 1.16)	I-309/CCL1 (3.94 ± 1.11)	MDC/CCL22 (2.76 ± 1.16)
MIP-1Α/CCL3 (3.54 ± 1.19)	IL-16 (3.44 ± 1.10)	Eotaxin-3/CCL26 (3.87 ± 1.10)	MIP-3Β/CCL19 (2.75 ± 1.14)
GM-CSF (3.50 ± 1.16)	Eotaxin-3/CCL26 (3.41 ± 1.08)	MIF (3.85 ± 1.04)	IL-16 (2.74 ± 1.04)

EASI: Eczema Area and Severity Index; MSE: mean squared error; SCORAD: SCORing Atopic Dermatitis; VAS: Visual analogue scale.

## 4. Discussion

In this study, we found that serum biomarkers can be used to diagnose mild-moderate AD patients with high AUC (0.85 ± 0.10) and accuracy (0.81 ± 0.15). The total IgE level was revealed to be the most essential feature for the classification analysis, reconfirming the key role of IgE, even in patients with mild-moderate AD. IgE plays a central role in allergen-induced inflammatory processes in various atopic diseases and presents a viable target for therapy [21]. AD is often associated with the elevated levels of serum IgE. However, it has also been reported that serum IgE is only weakly correlated with AD severity, which indicates the differential role in AD pathogenesis. When categorizing AD into intrinsic and extrinsic phenotypes, intrinsic AD shows normal serum levels of IgE and a relatively preserved skin barrier function, while the extrinsic AD presents with high levels of serum IgE with skin barrier dysfunction [21]. Through machine learning, we confirmed that IgE plays an important role in the diagnosis and severity of AD patients, but at the same time, we also found that the measurement of IgE alone is not sufficient to develop an adequate predictive model. Because the Th2 and Th22 immune pathways predominate in the AD inflammatory response, these biomarker candidates include Th2- and Th22-associated cytokines and chemokines including TARC/CCL17 and MDC/CCL22. Among the identified biomarkers in AD so far, TARC/CCL17 has shown the good evidence as an AD biomarker, and it was reported to be correlated with disease severity in both children and adults [4,9,22,23]. However, the levels of TARC/CCL17 varied among AD patients in similar disease severity [24], whereas some severe AD patients showed normal or low levels of serum TARC/CCL17, indicating the complexity of AD pathogenesis [22]. We also found that TARC/CCL17 contributes to the identification of patients with AD. A recent study showed that present AD biomarkers are mostly elevated in patients with moderate to severe but not mild AD patients [25]. Because our study included patients with mild to moderate AD, it may differ from previous studies that mainly focused on patients with moderate to severe AD. Considering that the proportion of patients with mild AD is as high as 27–67% of all patients [16], our results may reflect a more realistic clinical features. Additionally, some variables that did not show significant differences between groups according to univariate analysis were ranked as highly important in our models (e.g., MIF, IP-10/CXCL10).

Another strength of this study is that more diverse clinical outcomes were predicted than in previous research [9,26]. We found that classifiers trained on the same data failed to predict subjective itch ratings, whereas other AD-related scores (total SCORAD, objective SCORAD, and EASI scores) were predicted with moderate accuracy, and the most important features were different for each model. In addition, we found that state symptoms might be difficult to be predicted because of fluctuations of symptoms and effects of uncontrolled environmental and cognitive factors during collection of VAS ratings. Further studies are necessary to find the best variables and models to predict state factors and to investigate the role of Gro-α/CXCL1, MCP-4/CCL13, MDC/CCL22, MIF, IL-16 and their mechanisms, which ranked in top 10 in all significant prediction models.

The study has a number of limitations. First, the sample size is small and we did not validate our models with a new dataset. The model’s generalizability was limited by the inclusion of only AD patients with mild symptoms. Replication in an additional cohort, with patients with a wider range of AD severity, is required to confirm our results.

### 4.1 Future directions

**Fig 3:**
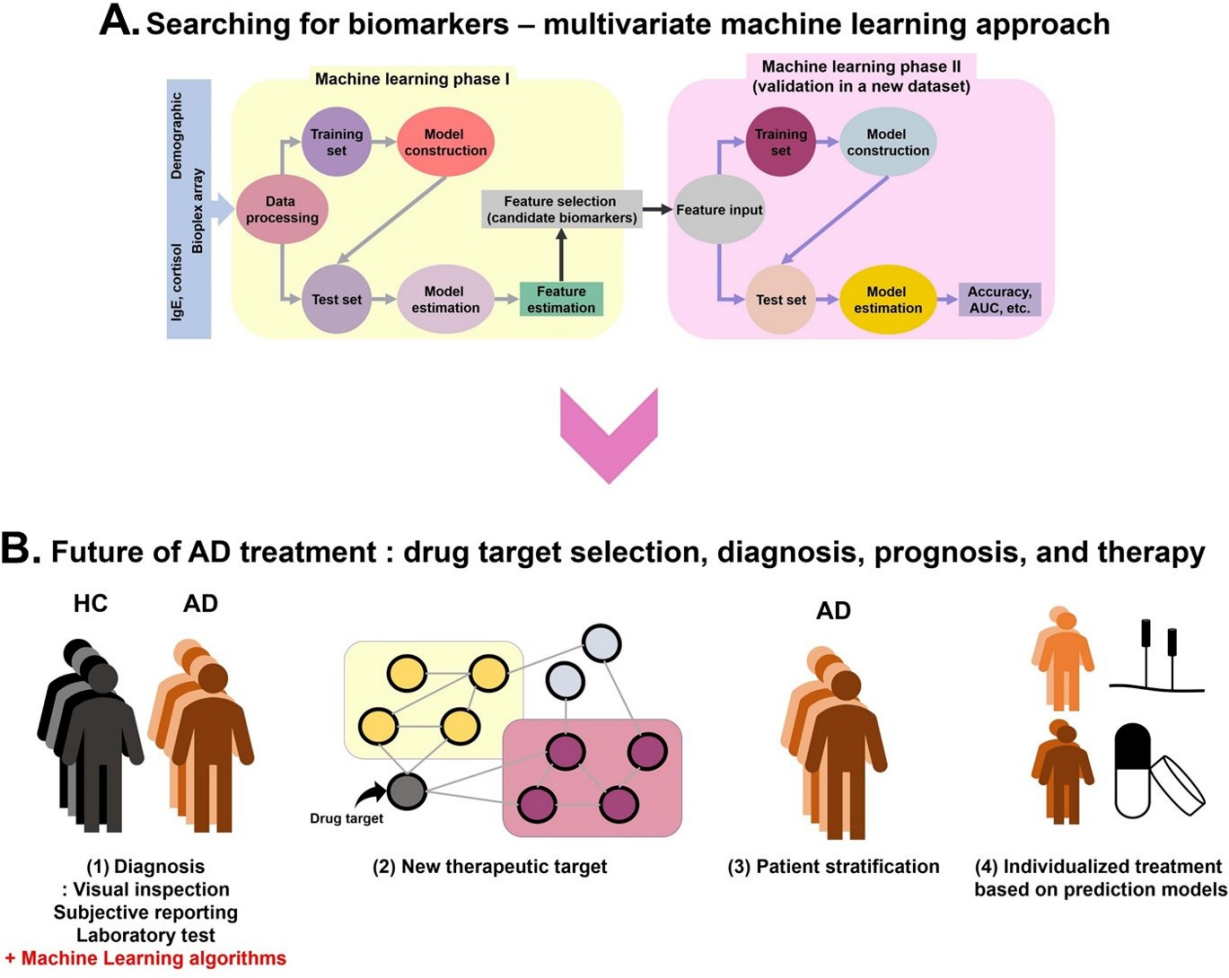
Workflow of machine learning-based approach for atopic dermatitis. (A) Current state of research and treatment of atopic dermatitis, (B) searching for biomarkers and prediction model development using multivariate machine learning, and (C) future of machine learning approach, improvement in drug target selection, diagnosis, prognosis, and individualized treatment. Currently, diagnosis of AD patients from HC is based on visual inspection, subjective reporting, and laboratory tests. Patients receive conventional therapies that are available for patients in consideration of symptoms, co-morbidities, co-medications, and costs. In the future, as multivariate machine learning techniques became possible, AD patients will receive early diagnosis and individualized treatment based on stratified types of disease and treatment-response prediction results using individual patterns of biomarkers. Various drug targets will be identified and evaluated based on the pathological pathway of candidate biomarkers of AD, which will expand the range of therapies for treatment of AD. Although given a wider range of treatment options, physicians can determine the best treatment strategy for each patient with the help of machine learning algorithms, which will eventually reduce healthcare costs and patients suffering. AD: atopic dermatitis; AUC: Area Under the Curve; HC: healthy controls.

We modeled symptom severity and group (AD vs. HC) as dependent variables in our machine learning model (Fig 3A), however, there are more various dependent and independent variables we should consider in AD population. Previous studies showed the effects of ethnicity, sex, age, disease duration, and comorbidities on AD symptoms and progression. Asian patients with AD, for example, have immune dysregulation that is intermediate between European-American AD and psoriasis, and this correlates well with the clinical phenomenology of Asian AD, which is marked by relatively clearly delineated psoriasiform lesions [27]. Th2 linked biomarkers (IL-5, IL-13, CCL13, CCL18, CCL26), IgE, and IL-22 are lower in elderly patients, whereas Th1 and Th17 related biomarkers are higher [28].

A multivariate machine learning approach could provide new insights and help validate the mechanisms of disease and biomarkers. It could also aid the development of new drugs targeting biomarker-related pathways. The best model and its parameters can be used with human patients to improve diagnosis and patient subtyping, and to objectively predict and assess treatment responses and disease progression. For example, candidate biomarkers identified by machine learning methods can become new therapeutic targets, and eventually, their influence will improve diagnosis (e.g., increase accuracy) and enable severity, treatment response, and prognosis prediction, as well as risk assessment, in the future. For example, if a model can forecast the magnitude of a treatment effect (e.g. changes of itch induced by a treatment, it would also advance research into the mechanism of the intervention. Given that this approach has made significant contributions to the therapy of many diseases, including cancer [29], neurodegenerative diseases [30], and pain [31], the final goal of this approach is to develop novel therapeutics targeting different pathways tailored for each patient. Such an improved prognostic test would be able to identify who would and who would not benefit from a new treatment in advance (Fig 3B). To achieve this goal, more variables might be preferable than using fewer variables to enhance the prediction performance of models, although we mainly focused on biomarkers collected from blood sample in this study. In future study, skin biopsy, genetic factors, physiological measurements, neuroimaging data, and microbiological properties can be integrated.

## 5. Conclusions

In summary, we established and assessed the multivariate prediction models and identified important serum features of mild-moderate AD patients. Our models showed moderate to high prediction performances, and we found potential serum biomarkers, e.g., IgE, CTACK/CCL27, TARC/CCL17, and MIF, contributing significantly to the diagnosis and severity prediction models. Our findings might contribute to a better understanding and personalized prevention and therapy of AD.

## Supporting information

S1 Table Comparison of serum cytokines levels between atopic dermatitis (AD) patients and healthy controls. (DOCX)
