# Correction: Prediction of disease severity using serum biomarkers in patients with mild-moderate Atopic Dermatitis: A pilot study

**DOI:** 10.1371/journal.pone.0296370

**Published:** 2023-12-20

**Authors:** In-Seon Lee, Mijung Yeom, Kyuseok Kim, Dae-Hyun Hahm, SeHyun Kang, Hi-Joon Park

There is an error in the Funding statement. The correct Funding statement is as follows: This work was supported by the National Research Foundation of Korea funded by the Korean government (NRF-2020R1A4A1018598, NRF-2021R1A2C2006818). The funders had no role in study design, data collection and analysis, decision to publish, or preparation of the manuscript. The authors received no specific funding for this work.
