# Supplementary material for: Prediction of disease severity using serum biomarkers in patients with mild-moderate Atopic Dermatitis: A pilot study
Source: PLoS One. 2023 Nov 2;18(11):e0293332. doi: 10.1371/journal.pone.0293332 (PMC10621918; doi:10.1371/journal.pone.0293332)
Supplement: S1 Table — (DOCX) [file pone.0293332.s001.docx]

**S1 Table.** Comparison of serum cytokines levels between atopic dermatitis (AD) patients and healthy controls

| Cytokines/  Chemokines (pg/ml) | AD patients (*n* = 28) | |  | Healthy controls (*n* = 20) | | FDR-adjusted  *p* value |
| --- | --- | --- | --- | --- | --- | --- |
|  | **mean (SD)** | **95% CI** |  | **mean (SD)** | **95% CI** |  |
| 6Ckine/CCL21 | 6194.81 (1653.96) | 5553.47-6836.15 |  | 5584.53 (987.07) | 5122.56-6046.49 | 0.16 |
| BCA-1/CXCL13 | 42.1 (41.02) | 26.19-58.01 |  | 33.25 (43.44) | 12.92-53.59 | 0.02* |
| CTACK/CCL27 | 1626.51 (540.33) | 1416.99-1836.03 |  | 1101.45 (252.12) | 983.46-1219.45 | <0.001*** |
| ENA-78/CXCL5 | 1521.8 (551.42) | 1307.98-1735.62 |  | 1074.15 (359.31) | 905.98-1242.31 | 0.018* |
| Eotaxin/CCL11 | 100.76 (33.07) | 87.93-113.58 |  | 79.14 (21.21) | 69.21-89.07 | 0.01* |
| Eotaxin-2/CCL24 | 708.06 (315.2) | 585.84-830.28 |  | 558.28 (184.69) | 471.84-644.72 | 0.1001 |
| Eotaxin-3/CCL26 | 15.02 (6.85) | 12.36-17.67 |  | 10.02 (4.62) | 7.86-12.18 | 0.01* |
| Fractalkine/CX3CL1 | 362.28 (204.13) | 283.13-441.43 |  | 228.14 (85.85) | 187.96-268.31 | 0.007** |
| GCP-2/CXCL6 | 63.32 (19.81) | 55.63-71 |  | 48.33 (20.19) | 38.89-57.78 | 0.0512 |
| GM-CSF | 182.06 (89.32) | 146.73-217.39 |  | 114.64 (54.06) | 89.34-139.94 | 0.008** |
| Gro-α/CXCL1 | 411.21 (76.26) | 381.64-440.78 |  | 343.59 (83.93) | 304.31-382.87 | 0.018* |
| Gro-β/CXCL2 | 414.35 (201.82) | 336.09-492.6 |  | 370.09 (165.56) | 292.6-447.58 | 0.38 |
| I-309/CCL1 | 86.91 (16.53) | 80.5-93.32 |  | 73 (13.29) | 66.78-79.22 | 0.018* |
| IFN-γ | 101.79 (41.17) | 85.82-117.75 |  | 69.22 (25.4) | 57.33-81.1 | 0.005** |
| IL-1β | 4.49 (1.9) | 3.75-5.23 |  | 3.1 (1.32) | 2.48-3.72 | 0.018* |
| IL-2 | 18.79 (8.69) | 15.43-22.16 |  | 12.54 (4.52) | 10.42-14.65 | 0.007** |
| IL-4 | 39.93 (9.46) | 36.26-43.6 |  | 33.6 (5.9) | 30.84-36.36 | 0.026* |
| IL-6 | 12.31 (5.75) | 10.08-14.54 |  | 8.01 (3.9) | 6.19-9.84 | 0.018** |
| IL-8/CXCL8 | 14.23 (5.29) | 12.18-16.28 |  | 11.3 (2.93) | 9.93-12.67 | 0.0512 |
| IL-10 | 57.08 (25.58) | 47.16-67 |  | 36.18 (15.42) | 28.96-43.4 | 0.005** |
| IL-16 | 888.48 (381.93) | 740.39-1036.58 |  | 713.07 (258.89) | 591.9-834.23 | 0.11 |
| IP-10/CXCL10 | 153.14 (52.5) | 132.79-173.5 |  | 148.96 (36.28) | 131.98-165.94 | 0.66 |
| I-TAC/CXCL11 | 14.93 (4.77) | 13.09-16.78 |  | 17.08 (10.8) | 12.03-22.14 | 0.45 |
| MCP-1/CCL2 | 55.97 (17.85) | 49.04-62.89 |  | 50.73 (14.15) | 44.11-57.35 | 0.25 |
| MCP-2/CCL8 | 75.43 (30.39) | 63.65-87.22 |  | 70.8 (24.34) | 59.41-82.19 | 0.648 |
| MCP-3/CCL7 | 214.22 (101.67) | 174.8-253.65 |  | 133.54 (58.23) | 106.28-160.79 | 0.005** |
| MCP-4/CCL13 | 106.05 (62.58) | 81.79-130.32 |  | 76.88 (54.7) | 51.28-102.48 | 0.02* |
| MDC/CCL22 | 1275.74 (539.23) | 1066.65-1484.83 |  | 935.14 (271.53) | 808.06-1062.22 | 0.03* |
| MIF | 743.1 (262.97) | 641.13-845.07 |  | 705.76 (370.78) | 532.23-879.29 | 0.749 |
| MIG/CXCL9 | 587.87 (324.61) | 462-713.74 |  | 361.98 (136.02) | 298.32-425.64 | 0.005** |
| MIP-1α/CCL3 | 9.03 (6.53) | 6.5-11.56 |  | 6.66 (1.64) | 5.89-7.42 | 0.03* |
| MIP-1δ/CCL15 | 9428.74 (6384.58) | 6903.09-11954.4 |  | 6206.18 (2555.32) | 5010.25-7402.1 | 0.13 |
| MIP-3α/CCL20 | 15.54 (7.85) | 12.5-18.59 |  | 14.91 (8.17) | 11.09-18.73 | 0.38 |
| MIP-3β/CCL19 | 596.07 (563.37) | 377.62-814.53 |  | 269.03 (161.82) | 193.3-344.76 | 0.005** |
| MPIF-1/CCL23 | 252.8 (128.27) | 203.07-302.54 |  | 263.07 (136.49) | 199.19-326.95 | 0.61 |
| SCYB16/CXCL16 | 503.44 (120.44) | 456.74-550.15 |  | 475.76 (126.01) | 416.79-534.74 | 0.531 |
| SDF-1α+β/CXCL12 | 6200.73 (2281.69) | 5315.98-7085.48 |  | 5959.5 (1959.16) | 5042.58-6876.41 | 0.749 |
| TARC/CCL17 | 246.09 (230.65) | 156.65-335.53 |  | 112.68 (59.38) | 84.89-140.47 | 0.002** |
| TECK/CCL25 | 614.69 (271.41) | 509.45-719.93 |  | 428.25 (126.35) | 369.11-487.38 | 0.007** |
| TNF-α | 33.78 (9.96) | 29.91-37.64 |  | 25.01 (6.44) | 22-28.02 | 0.018* |

Results are presented as Mean, standard deviation (SD) and 95% confidence intervals (CI). False Discovery Rate (FDR)-adjusted *p* values were calculated based on *p* values estimated by unpaired t test and Mann-Whitney test as appropriate. **p* ≤ 0.05, ***p* ≤ 0.01, and ****p* ≤ 0.001. AD, atopic dermatitis.
